# Supplementary material for: Assessment of the drugability of initial malaria infection through miniaturized sporozoite assays and high-throughput screening
Source: Commun Biol. 2023 Feb 23;6:216. doi: 10.1038/s42003-023-04599-3 (PMC9950425; doi:10.1038/s42003-023-04599-3)
Supplement: Supplementary file 2 — Supplementary Information [file 42003_2023_4599_MOESM2_ESM.pdf]

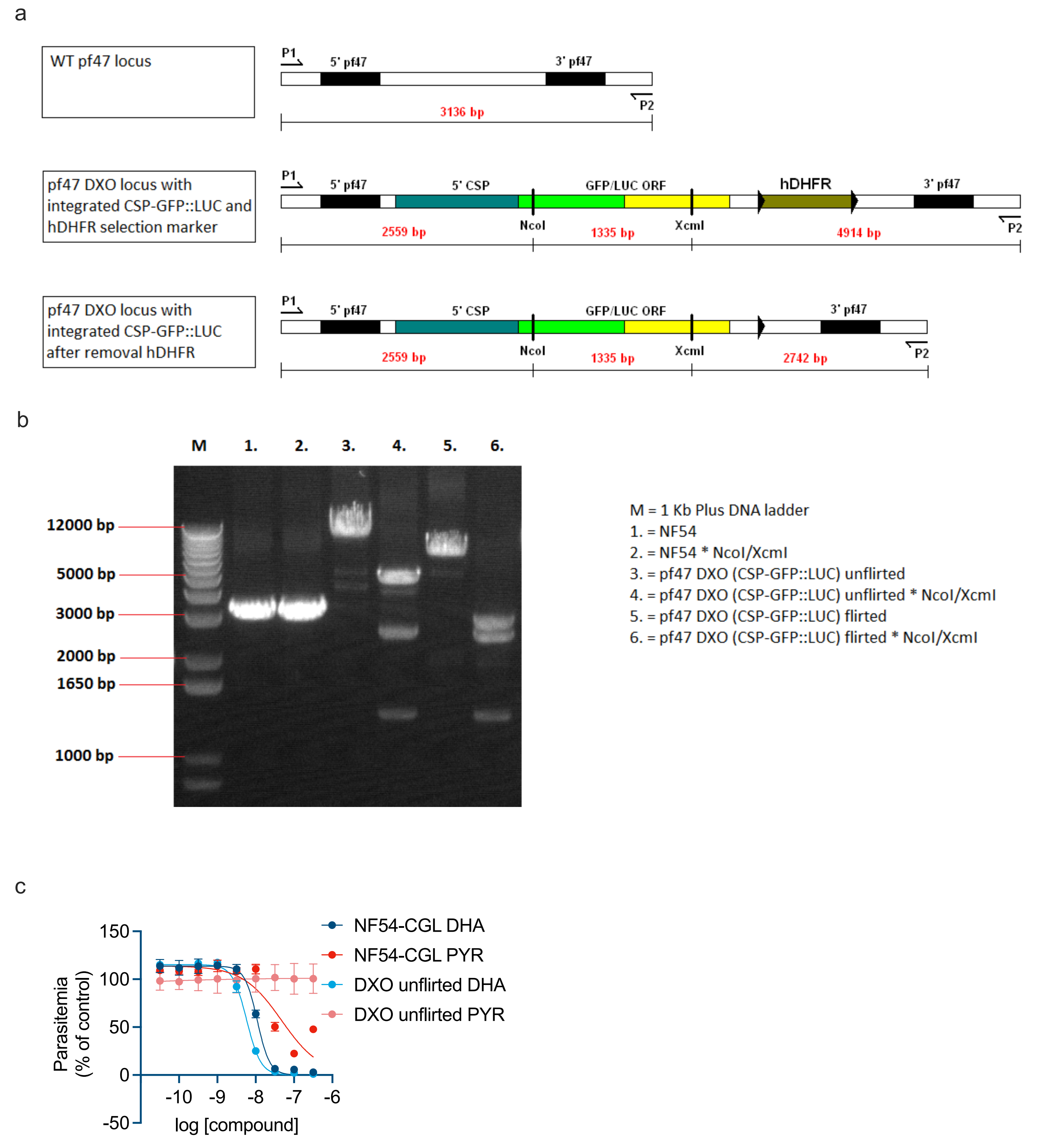

Supplemental Figure 1. Construction of the NF54 CGL reporter strain. a) schematic of the expected sizes of PCR fragements amplified with primers P1 and P2 indicated in the figure before and after NcoI/XcmI restriction digestion. b) Confirmation of succesful integration of the reporter cassette and excision of the hDHFR selection marker following FLP-mediated recombination. The figure shows an uncropped gel electrophoresis image of PCR products before and after NcoI/XcmI restriction digestion amplified from wildtype parasites (NF54) or cloned transfectants (DXO) before ('unflirted') and after ('flirted') FLP-mediated excision of the hDHFR selection cassette. c) sensitivity of unflirted double crossover (DXO) clone and the final reporter line NF54-CGL to dihydroartemisinin (DHA) and antifolate pyrimethamine (PYR), showing succesful excision of the hDHFR resistance gene in the final NF54-CGL strain. The figure shows results from an asexual blood stage replication assay in response to serial dilution of test compounds. Data were normalized to vehicle (0.1% DMSO) and 10 µM DHA controls. The figure shows averages and standard deviations from three replicate measurements

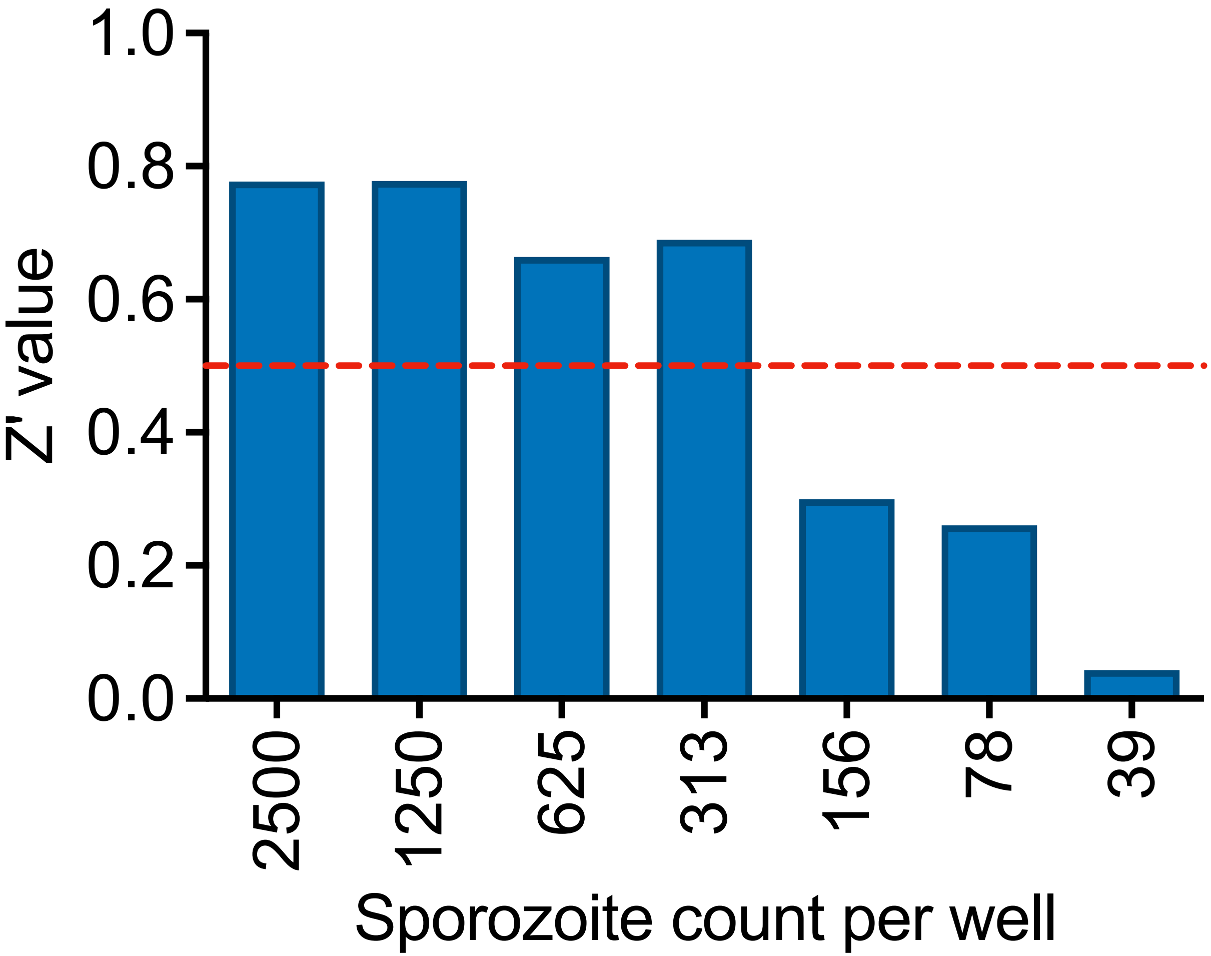

Supplemental Figure 2. Z’ in the sporozoite viability assay as a function of sporozoite number. Salivary gland sporozoites from reporter strain NF54-CGL were seeded in 384 well plates at the densities indicated in the figure and treated with vehicle (0.1% DMSO) or 1 μM gramicidin. Following 24 hour incubation, luciferase activity was determined and Z’ values were calculated based on 6 replicates for each condition.

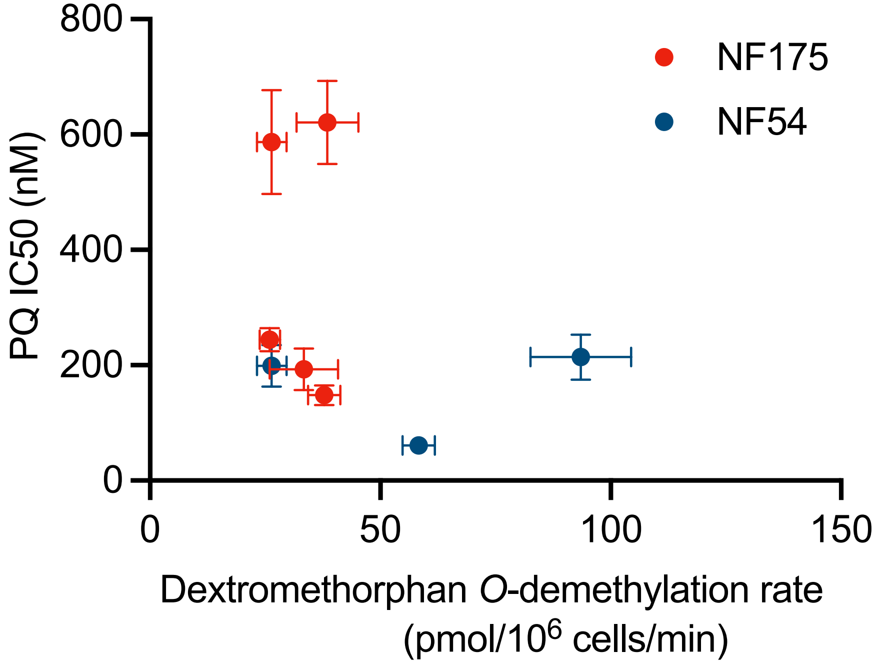

Supplemental Figure 3. Primaquine IC50 values in relation to CYP2D6 enzymatic activity in primary human hepatocytes. Primaquine activities against developing liver stage parasites from *P. falciparum* strain NF54 or NF175 were determined as described in the body text. These values are plotted against CYP2D6 activity as determined in dextromethorphan *O*-demethylation rate assays as provided by the supplier of the cells (Tebu-Bio). The figure shows averages and standard deviations from triplicate (primaquine IC50s) and duplicate (CYP2D6 activity) measurements.

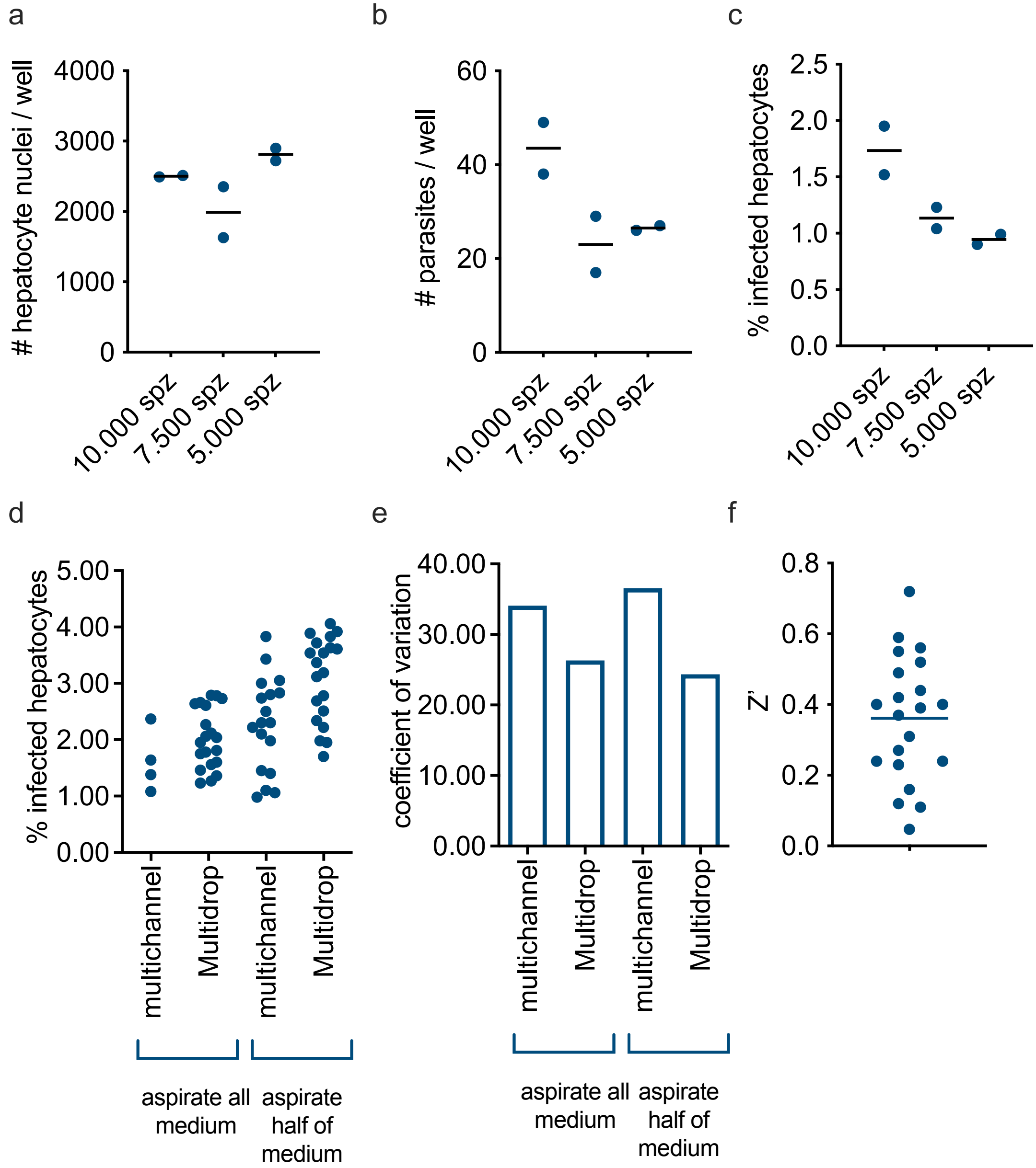

Supplemental Figure 4. Optimization of *P. falciparum* liver stage assays. Primary human hepatocytes were infected with salivary gland sporozoites from strain NF175 as described in the body text. a) number of hepatocyte nuclei as a function of sporozoite number. b) number of hsp70 positive parasites as a function of sporozoite number. c) percentage infected hepatocytes as a function of sporozoite number. Data in panels A-C is from 2 replicate experiments, bars indicate averages. d) Effect of manual (multichannel) or automated (multidrop) dispensing on % infected hepatocytes using scenario's where all medium was replaced during daily medium refreshments during the 4 day incubation period, or a scenario where half of the medium was replaced. Symbols indicate data from individual wells. e) coefficient of variation for the scenario's described under (d). f) Z' calculated from vehicle (0.1% DMSO) and 100 nM atovaquone controls. Each symbol indicates and independent experiment with 4 wells for each control.
