# Supplementary material for: Assessment of the drugability of initial malaria infection through miniaturized sporozoite assays and high-throughput screening
Source: Commun Biol. 2023 Feb 23;6:216. doi: 10.1038/s42003-023-04599-3 (PMC9950425; doi:10.1038/s42003-023-04599-3)
Supplement: Supplementary file 3 — Description of Additional Supplementary Files [file 42003_2023_4599_MOESM3_ESM.pdf]

Description of Additional Supplementary Files

File Name: Supplementary Data 1
Description: The source data behind figures in the paper.
